# Event-Triggered Distributed Fusion Estimator for Asynchronous Markov Jump Systems with Correlated Noises and Fading Measurements

**DOI:** 10.3390/s24020336

**Published:** 2024-01-05

**Authors:** Rui Zhang, Honglei Lin

**Affiliations:** School of Electronic Engineering, Heilongjiang University, Harbin 150080, China; zhangrui_hb@163.com

**Keywords:** asynchronous Markov jump system, distributed fusion, correlated noise, event-triggered, fading measurement

## Abstract

In this study, we investigate event-triggered distributed fusion estimation for asynchronous Markov jump systems subject to correlated noises and fading measurements. The measurement noises are interrelated, and they are simultaneously coupled with the system noise. The sensor samples measurements uniformly, and the sampling rates of the sensors are different. First, the asynchronous system is synchronized at state update points; then, the local filter is obtained. Furthermore, a variance-based event-triggered strategy is introduced between the local estimator and the fusion center to decrease the energy consumption of network communication. Then, a distributed fusion estimation algorithm is proposed using a matrix-weighted fusion criterion. Finally, the effectiveness of the algorithm is verified using computer simulations.

## 1. Introduction

With the development of network systems and sensor technologies, state estimation based on single sensor data usually cannot meet practical engineering requirements; thus, multi-sensor information fusion estimation has become a research focal point due to its reliability in terms of higher estimation accuracy. Furthermore, multiple sensors may have different sampling rates based on their requirements, and multi-rate sampling schemes are widely used in many systems, such as global positioning and inertial navigation systems [1]. Multi-rate multi-sensor information fusion estimation has a wide range of applications in many fields, such as target tracking and location, signal processing [2,3,4], and fault diagnosis [5,6]. Due to the limitations of communication resources and network bandwidths, an event-triggered data transmission strategy is introduced into state estimation to ensure state estimation accuracy while saving energy consumption. Moreover, networked uncertainties—including packet losses, fading measurements (FMs), random delays [7], and unknown parameter perturbations, which are usually described via Markov jump parameters—are inevitably considered in state estimation to improve the reliability and accuracy of state estimation results. Thus, the state fusion estimation problem is raised for multi-rate multi-sensor systems.

For multi-sensor information fusion estimation, the two main methods are centralized fusion and distributed fusion. Centralized fusion estimation offers global optimal estimation accuracy, but it is not convenient for sensor fault diagnosis and separation. Distributed fusion estimation (DFE) exhibits global suboptimality; however, it offers good robustness and stability, which has been widely investigated and applied, such as in signal processing [8], damage detection [9], and gas leak detection [10]. In [10], a weighted fusion algorithm is used to fuse multi-sensor data based on the distance between the sensor and the virtual leak source. In [11], the DFE problem is proposed for a multi-rate multi-sensor system in which the sensors have different sampling rates and are subject to correlated noises (CNs). Moreover, the iterative state-equation method is used to synchronize the multi-rate system, and the stability of the proposed algorithm is proven. In [12], the DFE for multi-rate systems is addressed, wherein the multiplicative noises in the observation equation are considered. Furthermore, the DFE algorithm is investigated for multi-rate systems with delays and FMs in [13]. In [14], the DFE algorithm with different sensor sampling rates in dynamic systems is studied, in which measurement noises are correlated with previous system noises. In [15], a state filter is processed for a fault detection system with multi-rate sampling, using lifting technology, and the phenomena of FMs and randomly occurring faults are investigated. In addition, a system may be affected by changes in the internal structure or environment during practical application, which can be described via a stochastic hybrid system.

Stochastic hybrid systems have attracted wide attention because of their strong practical background and their applications in some fields, such as power and computer control systems, as well as robotic rehabilitation [16,17]. Markov jump systems (MJSs) are frequently employed to simulate systems subjected to random changes in practice. In [18], it is proven that, if a Markov chain for a discrete MJS is ergodic and the system is mean-square stable, then the filter is stationary. In [19], the maximum likelihood method is used to solve the uncertain mode jumping problem of MJSs, and the state estimation algorithm is derived. In [20], the stability of networked control systems with packet losses is demonstrated. The stability of arbitrary packet loss is proven using a Lyapunov function; furthermore, the stability of Markov packet loss is proven based on the stability theory of MJSs. In [21], the state estimation problem for MJSs is investigated, in which the round-robin and weighted try-once-discard protocol are used to reduce the communication burden, and the hidden Markov model is used to solve a deception attack problem. In recent years, fusion estimation for asynchronous Markov jump systems (AMJSs) has attracted widespread attention, as it is difficult to unify the sensor sampling rate in sensor networks. In [22], asynchronous sensor measurements are processed via the batch process method for AMJSs subject to multiplicative noise, and the filter stationary condition is discussed. In [23], a resilient asynchronous estimator is designed for Markov jump neural networks, which ensures the stochastic stability of the system. In [24], a Markov-chain-driven network control system is investigated, in which the multi-rate control technology is used to improve dynamic control performance. However, there are a few state estimation approaches for AMJSs that consider resource constraints.

Considering the resource limitations of a network, traditional time-triggered mechanisms often cannot address the requirements of the system. Therefore, researchers have introduced event-triggered (ET) mechanisms and conducted numerous studies on them. In [25], a send-on-delta ET mechanism is adopted to reduce network traffic from sensor nodes. In [26], a DFE algorithm based on the ET mechanism is investigated, which improves the send-on-delta method by setting the trigger threshold based on the chi-square distribution. In [27], remote state estimation for linear systems based on the innovation ET mechanism is studied. In [28], a trigger mechanism, similar to the one investigated in [27], is used to investigate ET-based sequential fusion estimation for linear dynamic systems subject to CNs. Furthermore, based on [28], a noise estimator is proposed in [29] to enhance the accuracy of their systems, and a suboptimal estimator is also proposed to reduce the amount of computation. In [30], an ET-based state estimator for wireless sensor networks is proposed, which is affected by packet loss and CNs. In [31], a variance-based ET mechanism is studied, and the switching Riccati equation of its estimator can be calculated offline to determine whether to transmit the measurement data. An ET mechanism based on a normal distribution is proposed in [32] to obtain a better-performing triggered mechanism. In [33], a dissipative filter is studied under the ET mechanism for MJSs subject to time-varying delays. In [34], the variation in rotational inertia is modeled using a semi-Markov chain, and the ET mechanism is used to reduce the network bandwidth. In [35], an asynchronous sliding mode controller, based on the ET mechanism, is designed for singular MJSs. In [36], a fault detection filter for positive MJSs based on a dynamic ET mechanism is studied. The hidden Markov model is introduced to solve the asynchrony between the positive MJS and the fault detection filter. To date, fusion estimation for AMJSs based on the ET mechanism still requires development, especially considering the uncertainty problem in networking.

Motivated by the above discussions and based on our earlier work in [11], the DFE problem will be investigated for AMJSs with CNs and FMs based on the ET mechanism. Different from the distributed consensus Kalman filtering algorithm for sensor networks in [37], in this study, each sensor first generates the local filters (LFs) based on its own observation information. Then, the estimates from the LFs are sent to a remote fusion center (FC). Finally, all local estimators are further fused by using matrix-weighted fusion criteria [38] in the FC. The first step is to synchronize the multi-rate system to solve the asynchronous fusion estimation problem. Different from the iterating state equation in [11,12,13], the dummy measurement method in [39] is used to synchronize the system model to facilitate processing the Markov jump parameters, which reduces model complexity. Then, the state-space model is reestablished using the state augmentation method. Based on the equivalent state–space model, the optimal LFs are proposed in the linear minimum variance criterion. Afterward, the variance-based ET mechanism in [40] is introduced between the LFs and the FC to save network transmission resources. Furthermore, a matrix-weighted DFE algorithm is proposed. Different from ref. [22], in which a suboptimal fusion algorithm is proposed for the AMJSs with multiplicative noises, we can obtain an optimal matrix-weighted fusion estimation algorithm for AMJSs with CNs and FMs; moreover, the ET mechanism is considered from the LF to the FC, which can reduce redundant data transmission.

The contributions of this study are as follows:

(a) The model considers the various phenomena a system may encounter during application, including asynchronous uniform sampling, CNs, FMs, and Markov jump parameters, which can better reflect a real situation.
(b) From the LFs to the FC, a variance-based ET mechanism is designed, which can reduce energy consumption.
(c) An ET matrix-weighted DFE algorithm is proposed, which is more practical and energy-saving.

This article is organized as follows: The complex system is given in Section 2. A new, derived state–space model is presented in Section 3. LFs with the ET mechanism are presented in Section 4. The event-based DFE based on the matrix-weighted fusion criterion is presented in Section 5. Section 6 presents the simulated algorithm with an example. Finally, the conclusions are given in Section 7.

Notations: $R^{\bar{p}}$ represents the $\bar{p}$ -dimensional Euclidean space, $I_{\bar{p}}$ is the $\bar{p}$ -dimensional unit matrix, the transpose of matrix *B* is $B^{T}$ , the expectation of *B* is $E\left\{B\right\}$ , the covariance of *B* is $\mathrm{Cov}\left\{B\right\}$ , the spectral radius of matrix *B* is ρ(B) , $\delta_{kt}$ is the Kronecker delta function, and $1_{\left\{\cdot \right\}}$ is the Dirac measure. trB represents the trace of matrix *B*. Prob{A} denotes the occurrence probability of the event *A*. diag(•) denotes a block diagonal matrix. $col\{x_{1},\cdots ,x_{N}\}$ represents the column vector ${\left[\begin{array}{ccc} x_{1}^{T} & \ldots & x_{N}^{T} \end{array}\right]}^{T}$ .

## 2. Problem Formulation

Consider the following AMJSs with *L* sensors:

(1) $$x\left(\left(k+1\right)T\right)=\Phi_{\theta (kT)}x\left(kT\right)+\Gamma_{\theta (kT)}w\left(kT\right)$$

(2) $$y_{i}\left(n_{i}kT\right)=\beta_{i}\left(n_{i}kT\right)H_{i,\theta (n_{i}kT)}x\left(n_{i}kT\right)+G_{i,\theta (n_{i}kT)}v_{i}\left(n_{i}kT\right),\;i=1,2,\cdots ,L$$

where $x\left(kT\right)\in R^{\bar{p}}$ is the state of the system; $y_{i}\left(n_{i}kT\right)\in R^{q_{i}},\;i=1,2,\cdots ,L$ denotes the measurement of the sensor *i*; and $n_{i}kT$ is the measurement sampling period. $\omega \left(t\right)\in R^{r}$ and $v_{i}\left(n_{i}kT\right)\in R^{q_{i}},\;i=1,2,\cdots ,L$ are the process noise and measurement noises. $\Phi_{m}$ , $\theta_{m}$ , $H_{i,m}$ , and $G_{i,m},\;i=1,2,\cdots ,L;\;m=1,2,\cdots ,N$ are known constant matrices with appropriate dimensions. $\Phi_{m}$ satisfies the condition that, if $\rho (\Phi_{m})<1$ , then the system is stable. {θ(k)} is a discrete Markov chain with finite state space {1,2,⋯,N} .

$\beta_{i}\left(n_{i}kT\right),\;i=1,2\cdots ,L$ are the stochastic variables used to describe the FMs, which may occur due to aging or faults in the sensors. It takes values in $\left[a_{i}\left(n_{i}kT\right),b_{i}\left(n_{i}kT\right)\right]$ $\left(0\leq a_{i}\left(n_{i}kT\right)\leq b_{i}\left(n_{i}kT\right)\leq 1\right)$ with the expectation $E\left\{\beta_{i}\left(n_{i}kT\right)\right\}={\bar{\beta }}_{i}$ , the variance $\mathrm{Cov}\left\{\beta_{i}\left(n_{i}kT\right)\right\}=\sigma_{i}^{2}$ , and the probability distribution $p_{t}^{i}\left(c\right)$ .

Define $n_{i}=h_{x}/h_{i}\left(n_{i}\geq 1\right)$ as a positive integer, where $h_{x}$ and $h_{i}$ represent the state update and the measurement sampling rates, respectively. Without the loss of generality, the state update period *T* will be omitted in the later text. In addition, we also present the following four Assumptions.

**Assumption** **1.** *
ω(k) and $v_{i}\left(k\right)$ satisfy*

$$E\left\{\omega (k)\omega (\tau )\right\}=Q_{w}\delta_{k,\tau },\;E\left\{v_{i}\left(k\right)v_{j}\left(l\right)\right\}=R_{ij}\delta_{k,l},\;E\left\{\omega \left(\tau \right)v_{i}^{T}\left(l\right)\right\}=S_{wv_{i}}\delta_{\tau ,l}$$

*where $\tau =n_{i}k$ , $l=n_{j}k$ , and $Q_{w}\geq 0$ , $R_{ii}=R_{i}>0$ .*

**Assumption** **2.** *The initial state value x(0) is uncorrelated with ω(k) , $v_{i}\left(n_{i}k\right)$ , and*

$$E\left\{x(0)\right\}=\mu_{0},\;E\left\{(x\left(0\right)-\mu_{0}\right){\left(x\left(0\right)-\mu_{0}\right)}^{T}\}=P_{0}.$$

**Assumption** **3.** *The Markov chain {θ(k)} takes values in {1,2,⋯,N} with the transition probability matrix $p=[p_{nm}]$ , where $p_{nm}$ indicates the probability that the system will change from the n state to the m state, and the mode probability $\pi_{m}=Prob\left\{\theta \left(k\right)=m\right\}$ .*

**Assumption** **4.** *
$\beta_{i}\left(n_{i}k\right),i=1,2,\cdots ,L$ are uncorrelated with other variables.*

In consideration of Assumptions 1–4, in this study, we aim to propose a DFE algorithm for AMJSs (1) and (2) based on a variance-based ET mechanism.

## 3. Model Transformation

For Systems (1) and (2), the dummy measurement method [39] is used to transform the system into a synchronous sampling system; then, the following state–space model is obtained:

(3) $$x\left(k+1\right)=\Phi_{\theta (k)}x\left(k\right)+\Gamma_{\theta (k)}w\left(k\right)$$

(4) $$y_{i}\left(k\right)=\gamma_{i}\left(k\right)\left(\beta_{i}\left(k\right)H_{i,\theta (k)}x\left(k\right)+G_{i,\theta (k)}v_{i}\left(k\right)\right),\;i=1,2,\cdots ,L$$

where $\gamma_{i}\left(k\right)=\left\{\begin{array}{cc} 1 & k=n_{i}l,l=1,2,\cdots \\ 0 & k\neq n_{i}l \end{array}\right.$ .

If the *i*th sensor samples measurement data at time *k*, this means that $\gamma_{i}\left(k\right)=1$ . If the *i*th sensor does not sample measurement data at time *k*, this means that $\gamma_{i}\left(k\right)=0$ .

The Markov jump parameter is well suited for simulating stochastic systems with multiple sub-systems. The AMJSs based on (3) and (4) are augmented, and the estimate of x(k) is converted to the estimate of $x\left(k\right)1_{\left\{\theta (k)=m\right\}}$ . Denote $\varsigma \left(k\right)=col\{x\left(k\right)1_{\left\{\theta (k)=1\right\}},\cdots ,x\left(k\right)1_{\left\{\theta (k)=N\right\}}\}$ ; then, $x\left(k\right)=\sum_{m=1}^{N}\varsigma_{m}\left(k\right)$ , where $\varsigma_{m}\left(k\right)$ is the *m*th row of ς(k) . The augmented state–space model is given by the following:

(5) ς(k+1)=Aς(k)+Bw(k)

(6) $$y_{i}\left(k\right)=\gamma_{i}\left(k\right)\left(\beta_{i}\left(k\right)C_{i}\varsigma \left(k\right)+D_{i}v_{i}\left(k\right)\right),\;i=1,2,\cdots ,L$$

where

$$A=\left[\begin{array}{cccc} \Phi_{1}1_{\left\{\theta (k+1)=1|\theta (k)=1\right\}} & \Phi_{2}1_{\left\{\theta (k+1)=1|\theta (k)=2\right\}} & \cdots & \Phi_{N}1_{\left\{\theta (k+1)=1|\theta (k)=N\right\}}\\ \Phi_{1}1_{\left\{\theta (k+1)=2|\theta (k)=1\right\}} & \Phi_{2}1_{\left\{\theta (k+1)=2|\theta (k)=2\right\}} & \cdots & \Phi_{N}1_{\left\{\theta (k+1)=2|\theta (k)=N\right\}}\\ \vdots & \vdots & \vdots & \vdots \\ \Phi_{1}1_{\left\{\theta (k+1)=N|\theta (k)=1\right\}} & \Phi_{2}1_{\left\{\theta (k+1)=N|\theta (k)=2\right\}} & \cdots & \Phi_{N}1_{\left\{\theta (k+1)=N|\theta (k)=N\right\}} \end{array}\right]$$

$B=\left[\begin{array}{c} \left(\sum_{m=1}^{N}\Gamma_{m}1_{\left\{\theta (k)=m\right\}}\right)1_{\left\{\theta (k+1)=1\right\}}\\ \left(\sum_{m=1}^{N}\Gamma_{m}1_{\left\{\theta (k)=m\right\}}\right)1_{\left\{\theta (k+1)=2\right\}}\\ \vdots \\ \left(\sum_{m=1}^{N}\Gamma_{m}1_{\left\{\theta (k)=m\right\}}\right)1_{\left\{\theta (k+1)=N\right\}} \end{array}\right]$ , $C_{i}=\left[\begin{array}{ccc} H_{i,1} & \ldots & H_{i,N} \end{array}\right]$ , $D_{i}=\sum_{m=1}^{N}G_{i,m}1_{\left\{\theta (k)=m\right\}}$ .

Here, *A* is an N×N block matrix, *B* is an N×1 block matrix, $C_{i}$ is a 1×N block matrix, and $D_{i}$ is a 1×1 block matrix.

Furthermore, we define the following augmented matrices:

$$\bar{A}:=\left[\begin{array}{cccc} p_{11}\Phi_{1} & p_{21}\Phi_{2} & \ldots & p_{N1}\Phi_{N}\\ p_{12}\Phi_{1} & p_{22}\Phi_{2} & \ldots & p_{N2}\Phi_{N}\\ \vdots & \vdots & \vdots & \vdots \\ p_{1N}\Phi_{1} & p_{2N}\Phi_{2} & \ldots & p_{NN}\Phi_{N} \end{array}\right],\;\bar{B}:=\left[\begin{array}{c} \sum_{m=1}^{N}p_{m1}\pi_{m}\Gamma_{m}\\ \sum_{m=1}^{N}p_{m2}\pi_{m}\Gamma_{m}\\ \vdots \\ \sum_{m=1}^{N}p_{mN}\pi_{m}\Gamma_{m} \end{array}\right],$$

$${\bar{C}}_{i}:=\left[\begin{array}{ccc} H_{i,1} & \ldots & H_{i,N} \end{array}\right],{\bar{D}}_{i}:=\sum_{m=1}^{N}\pi_{m}G_{i,m},\;{\underline{D}}_{i}:=\sum_{m=1}^{N}\pi_{m}^{\frac{1}{2}}G_{i,m}$$

FMs reflect the degree of sensor aging and imperfect communication channels, which is a common phenomenon in sensor networks and generally occurs in the form of probability. Based on Assumptions 1–4, by transferring the multiplicative noise in Equation (6) to additive noise, we can obtain the following:

(7) $$y_{i}\left(k\right)=\gamma_{i}\left(k\right)\left(C_{i}^{*}\varsigma \left(k\right)+v_{i}^{*}\left(k\right)\right)$$

where $C_{i}^{*}={\bar{\beta }}_{i}C_{i}$ , $E\left\{C_{i}^{*}\right\}={\bar{C}}_{i}^{*}={\bar{\beta }}_{i}{\bar{C}}_{i}$ , $v_{i}^{*}\left(k\right)$ is given as follows:

(8) $$v_{i}^{*}\left(k\right)=\left(\beta_{i}\left(k\right)-{\bar{\beta }}_{i}\right)C_{i}\varsigma \left(k\right)+D_{i}v_{i}\left(k\right)$$

Then, the variance in $v_{i}^{*}\left(k\right)$ is as follows:

(9) $$Q_{v_{i}^{*}}\left(k\right)=E\left\{v_{i}^{*}\left(k\right){v_{i}^{*}}^{T}\left(k\right)\right\}=\sigma^{2}{\bar{C}}_{i}P\left(k\right){{\bar{C}}_{i}}^{T}+\sum_{m=1}^{N}\pi_{m}G_{i,m}R_{i}{G_{i,m}}^{T}$$

$P\left(k\right)=diag(P_{m}\left(k\right))$ is another form of the state second-moment matrix $P\left(k\right)=E\left\{\varsigma \left(k\right)\varsigma^{T}\left(k\right)\right\}$ , which can be calculated as follows:

(10) $$P_{m}\left(k\right)=E\left\{\varsigma_{m}\left(k\right){\varsigma_{m}}^{T}\left(k\right)\right\}=\sum_{n=1}^{N}p_{nm}\Phi_{n}P_{n}\left(k-1\right){\Phi_{n}}^{T}+\sum_{n=1}^{N}p_{nm}\pi_{n}\Gamma_{n}Q_{w_{i}}{\Gamma_{n}}^{T}$$

The process noise ω(k) has the following relationship with the new measurement noise $v_{i}^{*}\left(k\right)$ :

(11) $$S_{wv_{i}^{*}}=E\left\{\omega \left(k\right){v_{i}^{*}}^{T}\left(k\right)\right\}=S_{wv_{i}}{{\bar{D}}_{i}}^{T}$$

**Remark** **1.** *Up to now, AMJSs (1)–(2) with FMs are transformed to single-rate systems ((5) and (7)) with CNs. However, it is noteworthy that the parameters in the state Equation (5) and the new measurement Equation (7) are still uncertain, which is caused by Markov jump parameters. Thus, traditional Kalman filtering with CNs in [41] is no longer applicable. In addition, since FM phenomena are considered, the state second-moment matrix (10) must be derived to obtain the measurement noise variances (9).*

Next, we will propose the LEs based on the new state–space model ((5) and (7)).

## 4. Optimal Local Filters

In this section, the LFs will be derived under the ET mechanism. First, we will provide the LF without the ET; then, the variance-based ET mechanism will be introduced. Afterward, the LFs in the FC under the ET mechanism will be proposed.

### 4.1. Local Filters

**Theorem** **1.** *Under Assumptions 1–4, the state filter for Systems (5) and (7) based on Kalman filtering is given as follows:*

(12) $${\hat{\varsigma }}_{i}\left(k|k\right)={\hat{\varsigma }}_{i}\left(k|k-1\right)+K_{i}\left(k\right)\varepsilon_{i}\left(k\right)$$

*The innovation $\varepsilon_{i}\left(k\right)$ is calculated as follows:*

(13) $$\varepsilon_{i}\left(k\right)=y_{i}\left(k\right)-\gamma_{i}\left(k\right){\bar{C}}_{i}^{*}{\hat{\varsigma }}_{i}\left(k|k-1\right)$$

*Then, the variance in innovation is calculated as follows:*

(14) $$Q_{\varepsilon_{i}}\left(k\right)=E\left\{\varepsilon_{i}\left(k\right)\varepsilon_{i}^{T}\left(k\right)\right\}=\gamma_{i}\left(k\right)({\bar{C}}_{i}^{*}P_{\varsigma_{i}}\left(k|k-1\right){{\bar{C}}_{i}^{*}}^{T}+Q_{v_{i}^{*}}\left(k\right)$$

*where the gain matrix for the state filter can be written as follows:*

(15) $$K_{i}\left(k\right)=\gamma_{i}\left(k\right)P_{\varsigma_{i}}\left(k|k-1\right){{\bar{C}}_{i}^{*}}^{T}Q_{\varepsilon_{i}}^{-1}\left(k\right)$$

*The prediction for the state ${\hat{\varsigma }}_{i}\left(k+1|k\right)$ is as follows:*

(16) $${\hat{\varsigma }}_{i}\left(k+1|k\right)=\bar{A}{\hat{\varsigma }}_{i}\left(k|k\right)+\bar{B}{\hat{w}}_{i}\left(k|k\right)$$

*where the system noise filter is computed as follows:*

(17) $${\hat{w}}_{i}\left(k|k\right)=K_{w_{i}}\left(k\right)\varepsilon_{i}\left(k\right)$$

*The gain matrix for the system noise filter is computed via the following:*

(18) $$K_{w_{i}}\left(k\right)=\gamma_{i}\left(k\right)S_{wv_{i}^{*}}Q_{\varepsilon_{i}}^{-1}\left(k\right)$$

*The estimation error covariance matrix (EECM) for the state can be written as follows:*

(19) $$P_{\varsigma_{i}}\left(k|k\right)=E\left\{{\tilde{\varsigma }}_{i}\left(k|k\right){{\tilde{\varsigma }}_{i}}^{T}\left(k|k\right)\right\}=\left(I_{\bar{p}N}-K_{i}\left(k\right){\bar{C}}_{i}^{*}\right)P_{\varsigma_{i}}\left(k|k-1\right)$$

*The EECM for the system noise is calculated via the following:*

(20) $$P_{w_{i}}\left(k|k\right)=E\left\{{\tilde{w}}_{i}\left(k|k\right){{\tilde{w}}_{i}}^{T}\left(k|k\right)\right\}=Q_{w_{i}}-K_{w_{i}}\left(k\right)Q_{\varepsilon_{i}}\left(k\right){K_{w_{i}}}^{T}\left(k\right)$$

*Then, the EECM between the system noise and the state is calculated as follows:*

(21) $$P_{\varsigma w_{i}}\left(k|k\right)=E\left\{{\tilde{\varsigma }}_{i}\left(k|k\right){{\tilde{w}}_{i}}^{T}\left(k|k\right)\right\}=-P_{\varsigma_{i}}\left(k|k-1\right){{\bar{C}}_{i}^{*}}^{T}{K_{w_{i}}}^{T}\left(k\right)$$

*where the prediction EECM is computed using the following:*

(22) $$P_{\varsigma_{i}}\left(k+1|k\right)=E\left\{{\tilde{\varsigma }}_{i}\left(k+1|k\right){{\tilde{\varsigma }}_{i}}^{T}\left(k+1|k\right)\right\}=\bar{A}P_{\varsigma_{i}}\left(k|k\right){\bar{A}}^{T}+\bar{A}P_{\varsigma w_{i}}\left(k|k\right){\bar{B}}^{T}+\bar{B}{P_{\varsigma w_{i}}}^{T}\left(k|k\right){\bar{A}}^{T}\;+\bar{B}P_{w_{i}}\left(k|k\right){\bar{B}}^{T}+P\left(k+1\right)-\bar{A}P\left(k\right){\bar{A}}^{T}-\bar{B}Q_{w}{\bar{B}}^{T}$$

The initial value set ${\hat{\varsigma }}_{i}\left(0|-1\right)=\left[\begin{array}{c} \;\mu_{0}1_{\left\{\theta (0)=1\right\}}\\ \vdots \\ \mu_{0}1_{\left\{\theta (0)=N\right\}} \end{array}\right]$ , and $P_{\varsigma_{i}}\left(0|-1\right)=diag\left(P_{0}1_{\left\{\theta (0)=m\right\}}\right).$

**Proof.**  Formula (12) can be derived using the projection theory [42]. The innovation is defined as follows:

(23) $$\begin{array}{c} \varepsilon_{i}\left(k\right)=y_{i}\left(k\right)-{\hat{y}}_{i}\left(k|k-1\right)=y_{i}\left(k\right)-\gamma_{i}\left(k\right)({\bar{C}}_{i}^{*}{\tilde{\varsigma }}_{i}\left(k|k-1\right) \end{array}$$

Then, we obtain (13). By substituting (7) into (23), the innovation can be rewritten as follows:

(24) $$\begin{array}{c} \varepsilon_{i}\left(k\right)=\gamma_{i}\left(k\right)\left({\bar{C}}_{i}^{*}{\tilde{\varsigma }}_{i}\left(k|k-1\right)+v_{i}^{*}\left(k\right)\right) \end{array}$$

Substituting (24) into the variance $Q_{\varepsilon i}\left(k\right)=E\left\{\varepsilon_{i}\left(k\right){\varepsilon_{i}}^{T}\left(k\right)\right\}$ of the innovation yields (14).

From (24) and $\varsigma \left(k\right)={\tilde{\varsigma }}_{i}\left(k|k-1\right)+{\hat{\varsigma }}_{i}\left(k|k-1\right)$ , we have

(25) $$E\left\{\varsigma \left(k\right){\varepsilon_{i}}^{T}\left(k\right)\right\}=\gamma_{i}\left(k\right)P_{\varsigma i}\left(k|k-1\right){\bar{C}}_{i}^{*}$$

Substituting (25) and (14) into $K_{i}\left(k\right)=E\left\{\varsigma \left(k\right){\varepsilon_{i}}^{T}\left(k\right)\right\}{\left(Q_{\varepsilon_{i}}\left(k\right)\right)}^{-1}$ yields (15).

According to the projection theory [42], we have (16). From $E\{\omega \left(k\right)|y_{i}\left(0\right),\cdots ,y_{i}\left(k-1\right)\}=0$ , (17) can be computed.

By substituting (24) into $E\{w\left(k\right){\varepsilon_{i}}^{T}\left(k\right)\}$ , it follows that

(26) $$E\left\{w\left(k\right){\varepsilon_{i}}^{T}\left(k\right)\right\}=\gamma_{i}\left(k\right)S_{wv_{i}^{*}}{{\bar{D}}_{i}}^{T}$$

Substituting (26) into $K_{w_{i}}\left(k\right)=E\left\{w\left(k\right){\varepsilon_{i}}^{T}\left(k\right)\right\}Q_{\varepsilon_{i}}^{-1}\left(k\right)$ yields (18).

From the filter error equation ${\tilde{\varsigma }}_{i}\left(k|k\right)=\varsigma \left(k\right)-{\hat{\varsigma }}_{i}\left(k|k\right)$ and (12), we have

(27) $${\tilde{\varsigma }}_{i}\left(k|k\right)={\tilde{\varsigma }}_{i}\left(k|k-1\right)-\gamma_{i}\left(k\right)K_{i}\left(k\right)\varepsilon_{i}\left(k\right)$$

By substituting (27) into $P_{\varsigma_{i}}\left(k|k\right)=E\left\{{\tilde{\varsigma }}_{i}\left(k|k\right){{\tilde{\varsigma }}_{i}}^{T}\left(k|k\right)\right\}$ and then using (25), (15) and $E\left\{{\tilde{\varsigma }}_{i}\left(k|k-1\right){\varepsilon_{i}}^{T}\left(k\right)\right\}=E\left\{\varsigma_{i}\left(k\right){\varepsilon_{i}}^{T}\left(k\right)\right\}$ , it yields (19).

From the prediction error equation ${\tilde{\varsigma }}_{i}\left(k+1|k\right)=\varsigma \left(k+1\right)-{\hat{\varsigma }}_{i}\left(k+1|k\right)$ , (5), and (16), we can obtain that

(28) $${\tilde{\varsigma }}_{i}\left(k+1|k\right)=\varsigma \left(k+1\right)-{\hat{\varsigma }}_{i}\left(k+1|k\right)\;=A\varsigma \left(k\right)-\bar{A}\varsigma \left(k\right)+Bw\left(k\right)-\bar{B}w\left(k\right)+\bar{A}\varsigma \left(k\right)-\bar{A}{\tilde{\varsigma }}_{i}\left(k|k\right)+\bar{B}w\left(k\right)-\bar{B}{\hat{w}}_{i}\left(k|k\right)\;=\bar{A}{\tilde{\varsigma }}_{i}\left(k|k\right)+\bar{B}{\tilde{w}}_{i}\left(k|k\right)+\left(A-\bar{A}\right)\varsigma \left(k\right)+\left(B-\bar{B}\right)w\left(k\right)$$

Substituting (28) into $P_{\varsigma_{i}}\left(k+1|k\right)=E\left\{{\tilde{\varsigma }}_{i}\left(k+1|k\right){{\tilde{\varsigma }}_{i}}^{T}\left(k+1|k\right)\right\}$ yields (22), where

(29) $$E\left\{\left(A-\bar{A}\right)\varsigma \left(k\right)\varsigma^{T}\left(k\right){\left(A-\bar{A}\right)}^{T}\right\}=E\left\{A\varsigma \left(k\right)\varsigma^{T}\left(k\right)A^{T}\right\}-E\left\{\bar{A}\varsigma \left(k\right)\varsigma^{T}\left(k\right){\bar{A}}^{T}\right\}$$

(30) $$E\left\{\left(B-\bar{B}\right)\omega \left(k\right)\omega^{T}\left(k\right){\left(B-\bar{B}\right)}^{T}\right\}=E\left\{B\omega \left(k\right)\omega^{T}\left(k\right)B^{T}\right\}-E\left\{\bar{B}\omega \left(k\right)\omega^{T}\left(k\right){\bar{B}}^{T}\right\}$$

(31) $$P\left(k+1\right)=E\left\{A\varsigma \left(k\right)\varsigma^{T}\left(k\right)A^{T}\right\}+E\left\{B\omega \left(k\right)\omega^{T}\left(k\right)B^{T}\right\}$$

By substituting (17) into ${\tilde{\omega }}_{i}\left(k|k\right)=\omega \left(k\right)-{\hat{\omega }}_{i}\left(k|k\right)$ , another form of the white noise filtering error equation can be obtained as follows:

(32) $${\tilde{\omega }}_{i}\left(k|k\right)=\omega \left(k\right)-K_{\omega_{i}}\left(k\right)\varepsilon_{i}\left(k\right)$$

Substituting (32) into $P_{\omega_{i}}\left(k|k\right)=E\left\{{\tilde{\omega }}_{i}\left(k|k\right){{\tilde{\omega }}_{i}}^{T}\left(k|k\right)\right\}$ leads to (20), where $K_{w_{i}}\left(k\right)=E\left\{\omega \left(k\right){\varepsilon_{i}}^{T}\left(k\right)\right\}Q_{\varepsilon_{i}}^{-1}\left(k\right)$ are used.

Substituting (32) into $P_{\varsigma \omega_{i}}\left(k|k\right)=E\left\{{\tilde{\varsigma }}_{i}\left(k|k\right){{\tilde{\omega }}_{i}}^{T}\left(k|k\right)\right\}$ leads to (21), where $E\{{\hat{\varsigma }}_{i}\left(k|k\right){{\tilde{\omega }}_{i}}^{T}\left(k|k\right)\}=0$ , $E\{\varsigma \left(k\right)\omega^{T}\left(k\right)\}=0$ , and (25) are used in the derivation of this equality. Thus, the proof is completed.    □

**Remark** **2.** *Disregarding Markov jump parameters, it is noteworthy that the LF algorithm in Theorem 1 is asymptotically stable, which can be directly obtained by using the results in [11,43,44]. Moreover, in refs. [43,44], the asymptotically stable and period steady-state properties are proven based on the classical Kalman filtering theory, and the period is only the measurement sampling period for each local estimator. Compared with the iterative state-equation synchronization method in [11,43,44], the dummy measurement synchronization method is adopted in this study. This is because it can obtain a simpler state-space model at the state update points, making it more convenient to handle Markov jump parameters. Although the same estimation accuracy can be obtained in LF algorithms based on the two synchronization methods, the stability and convergence of our proposed estimation algorithm based on the dummy measurement synchronization method cannot reasonably be given at state update points where there are no measurement data. In future work, we will try to provide a solution to the stability problem using the iterative equation state method.*

Theorem 1 describes the LF when all sensor measurements are used. Compared with the classical Kalman filtering approach utilized in ref. [41], the derivation difficulty of the LF algorithm mainly lies in the state prediction EECM (22). Next, we will present the ET mechanism between the LFs and the FC. If the estimates of all sensors are transmitted, the computing storage of the FC will be increased; simultaneously, there will be significant unnecessary resource consumption in the process of data transmission. Therefore, the ET mechanism is introduced to reduce local transmission resources to the FC, thus decreasing resource storage.

### 4.2. Local Filters in Fusion Center

In this subsection, we introduce an ET mechanism to reduce redundant data transmission, thus maximizing the use of limited network resources. In [40], a variance-based ET mechanism is introduced between the LFs and the FC to determine whether data are transmitted.

**Figure 1:**
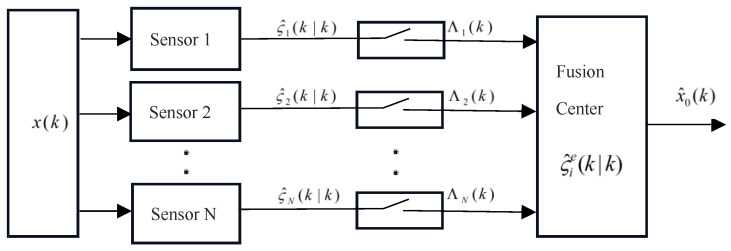
The structure of the estimation algorithm based on the variance-based ET mechanism.

The variance-based ET mechanism condition is defined as follows:

(33) $$\Lambda_{i}\left(k\right)=\left\{\begin{array}{cc} 0, & {trP}_{\varsigma_{i}}\left(k\right)-\theta_{i}<0\\ 1, & {trP}_{\varsigma_{i}}\left(k\right)-\theta_{i}\geq 0 \end{array}\right.$$

where $P_{\varsigma_{i}}\left(k\right)$ is the filtering EECM of the *i*th sensor. $\theta_{i}=trP_{d}+m$ is the upper bound of the DFE accuracy. trP is the trace of *P*. $P_{d}$ represents the optimal EECM of the matrix-weighted DFE, which can be obtained from [11]. *m* is a given threshold, where m≥0 means that the estimation accuracy is set to be less than or equal to the optimal fusion accuracy. When $\Lambda_{i}\left(k\right)=1$ , the FC can receive the LF date; when $\Lambda_{i}\left(k\right)=0$ , the FC cannot receive the LE, but the predicted value of the LFs stored in the FC at the previous time can be used. Figure 1 shows the structure of the proposed ET estimation algorithm.

**Theorem** **2.** *When the LF satisfies the ET condition at moment k, that is, $\Lambda_{i}\left(k\right)=1$ , the proposed filter degenerates to Theorem 1, and we have the following:*

(34) $${\hat{\varsigma }}_{i}^{e}\left(k|k\right)={\hat{\varsigma }}_{i}\left(k|k\right)$$

(35) $$P_{\varsigma_{i}}^{e}\left(k|k\right)=P_{\varsigma_{i}}\left(k|k\right)$$

*When the LF does not satisfy the ET transmission condition, that is, $\Lambda_{i}\left(k\right)=0$ , the FC receives the state LFs ${\hat{\varsigma }}_{i}^{e}\left(k|k\right)$ according to the one-step prediction ${\hat{\varsigma }}_{i}^{e}\left(k|k-1\right)$ of the state LFs ${\hat{\varsigma }}_{i}\left(k-1|k-1\right)$ stored in the FC at moment k−1 . Thus, there are two cases for the state filter.*

*(a)*  *If $\Lambda_{i}\left(k\right)=0$ and $\Lambda_{i}\left(k-1\right)=1$ , we have*
(36) $${\hat{\varsigma }}_{i}^{e}\left(k|k\right)=\bar{A}{\hat{\varsigma }}_{i}\left(k-1|k-1\right)+\bar{B}{\hat{w}}_{i}\left(k-1|k-1\right)$$
(37) $$P_{\varsigma_{i}}^{e}\left(k|k\right)=E\left\{{\tilde{\varsigma }}_{i}^{e}\left(k|k\right){{\tilde{\varsigma }}_{i}}^{eT}\left(k|k\right)\right\}=\bar{A}P_{\varsigma_{i}}\left(k-1|k-1\right){\bar{A}}^{T}+\bar{A}P_{\varsigma w_{i}}\left(k-1|k-1\right){\bar{B}}^{T}\;+\bar{B}{P_{\varsigma w_{i}}}^{T}\left(k-1|k-1\right){\bar{A}}^{T}+\bar{B}P_{w_{i}}\left(k-1|k-1\right){\bar{B}}^{T}+P\left(k\right)-\bar{A}P\left(k-1\right){\bar{A}}^{T}-\bar{B}Q_{w}{\bar{B}}^{T}$$
*(b)*  *If $\Lambda_{i}\left(k\right)=0$ and $\Lambda_{i}\left(k-1\right)=0$ , we have*
(38) $${\hat{\varsigma }}_{i}^{e}\left(k|k\right)=\bar{A}{\hat{\varsigma }}_{i}\left(k-1|k-1\right)$$
(39) $$P_{\varsigma_{i}}^{e}\left(k|k\right)=E\left\{{\tilde{\varsigma }}_{i}^{e}\left(k|k\right){{\tilde{\varsigma }}_{i}}^{eT}\left(k|k\right)\right\}=P\left(k\right)-\bar{A}P\left(k-1\right){\bar{A}}^{T}+\bar{A}P_{\varsigma_{i}}\left(k-1|k-1\right){\bar{A}}^{T}$$

**Proof.**  When $\Lambda_{i}\left(k\right)=0$ and $\Lambda_{i}\left(k-1\right)=1$ , based on the projection theorem [42], the state filter of the sensor *i* can be written as follows:

(40) $${\hat{\varsigma }}_{i}^{e}\left(k|k\right)={\hat{\varsigma }}_{i}^{e}\left(k|k-1\right)$$

The one-step predictor ${\hat{\varsigma }}_{i}^{e}\left(k|k-1\right)$ is calculated via

(41) $${\hat{\varsigma }}_{i}^{e}\left(k|k-1\right)=\bar{A}{\hat{\varsigma }}_{i}\left(k-1|k-1\right)+\bar{B}{\hat{w}}_{i}\left(k-1|k-1\right)$$

Thus, it follows (36). Based on ${\tilde{\varsigma }}_{i}^{e}\left(k|k\right)=\varsigma \left(k\right)-{\hat{\varsigma }}_{i}^{e}\left(k|k\right)$ , from (40), we have ${\tilde{\varsigma }}_{i}^{e}\left(k|k\right)={\tilde{\varsigma }}_{i}^{e}\left(k|k-1\right)$ ; then, substituting it into $P_{\varsigma_{i}}^{e}\left(k|k\right)=E\left\{{\tilde{\varsigma }}_{i}^{e}\left(k|k\right){{\tilde{\varsigma }}_{i}}^{eT}\left(k|k\right)\right\}$ yields (37).

When $\Lambda_{i}\left(k\right)=0$ and $\Lambda_{i}\left(k-1\right)=0$ , based on the projection theorem [42], we obtain the state filter as follows:

(42) $${\hat{\varsigma }}_{i}^{e}\left(k|k\right)={\hat{\varsigma }}_{i}^{e}\left(k|k-1\right)=\bar{A}{\hat{\varsigma }}_{i}\left(k-1|k-1\right)$$

The state estimation error equation is given via

(43) $${\tilde{\varsigma }}_{i}^{e}\left(k|k\right)=\left(A-\bar{A}\right)\varsigma \left(k-1\right)+\bar{A}{\tilde{\varsigma }}_{i}\left(k-1|k-1\right)+Bw\left(k-1\right)$$

By substituting (43) into $P_{\varsigma_{i}}^{e}\left(k|k\right)=E\left\{{\tilde{\varsigma }}_{i}^{e}\left(k|k\right){{\tilde{\varsigma }}_{i}}^{eT}\left(k|k\right)\right\}$ , we have (39). Thus, the proof is completed.    □

Based on Theorems 1 and 2, the LFs in the FC for Systems (1) and (2) are

(44) $${\hat{x}}_{i}\left(k|k\right)=\sum_{m=1}^{N}{\hat{\varsigma }}_{i,m}^{e}\left(k|k\right)$$

(45) $$P_{x_{i}}^{c}\left(k|k\right)=\sum_{m=1}^{N}\sum_{n=1}^{N}P_{i,mn}^{e}\left(k|k\right)$$

where ${\hat{\varsigma }}_{i,m}^{e}\left(k|k\right)$ is the *m*th rows of ${\hat{\varsigma }}_{i}^{e}\left(k|k\right)$ , and $P_{i,mn}^{e}\left(k|k\right)$ is the (n,m) th sub-blocks of $P_{\varsigma_{i}}^{e}\left(k|k\right)$ .

**Remark** **3.** *In this study, we assume that the FC can store the LFs at the previous moment, and at the initial moment, all sensors transmit data to the FC, that is, $\Lambda_{i}\left(0\right)=1,i=1,2,\cdots ,L$ .*

**Remark** **4.** *The selection range of the threshold is $\min\left\{trP_{\varsigma_{i}}\left(k\right)-trP_{d}\right\}<m<\max\left\{trP_{\varsigma_{i}}\left(k\right)-trP_{d}\right\}$ . When $m<\min\{trP_{\varsigma_{i}}\left(k\right)-trP_{d}\}$ , it means that all local estimation data can be transmitted to the FC.*

## 5. Event-Triggered Distributed Fusion Estimation Algorithm

This section mainly derives the estimation error cross-covariance matrices (EECCMs) between any two LFs under the variance-based ET mechanism and the DFE under the matrix-weighted fusion criterion. According to Theorems 1 and 2 in the previous section, the results can be obtained by using the following.

**Theorem** **3.** *Based on Assumptions 1–4 and Theorem 2, the EECCMs $P_{\varsigma_{i}\varsigma_{j}}\left(k\right)$ between sensor i and sensor j are calculated as follows:*

*The one-step prediction EECCMs are computed via*

(46) $$P_{\varsigma_{i}\varsigma_{j}}\left(k|k-1\right)=E\left\{{\tilde{\varsigma }}_{i}^{e}\left(k|k-1\right){{\tilde{\varsigma }}_{j}}^{eT}\left(k|k-1\right)\right\}=\bar{A}P_{\varsigma_{i}\varsigma_{j}}\left(k-1\right){\bar{A}}^{T}+\bar{A}P_{\varsigma_{i}w_{j}}\left(k-1\right){\bar{B}}^{T}+\bar{B}P_{\varsigma_{i}w_{j}}^{T}\left(k-1\right){\bar{A}}^{T}\;+\bar{B}P_{w_{i}w_{j}}\left(k-1\right){\bar{B}}^{T}+P\left(k\right)-\bar{A}P\left(k-1\right){\bar{A}}^{T}-\bar{B}Q_{w}{\bar{B}}^{T}$$

*The filtering EECCMs are calculated via*

(47) $$P_{\varsigma_{i}\varsigma_{j}}\left(k\right)=E\left\{{\tilde{\varsigma }}_{i}^{e}\left(k|k\right){{\tilde{\varsigma }}_{j}}^{eT}\left(k|k\right)\right\}=\left[I_{\bar{p}N}-\Lambda_{i}\left(k\right)K_{i}\left(k\right){\bar{C}}_{i}^{*}\right]P_{\varsigma_{i}\varsigma_{j}}\left(k|k-1\right){\left[I-\Lambda_{j}\left(k\right)K_{j}\left(k\right){\bar{C}}_{j}^{*}\right]}^{T}\;+\Lambda_{i}\left(k\right)\Lambda_{j}\left(k\right)K_{i}\left(k\right)R_{ij}{K_{j}}^{T}\left(k\right)$$

*The EECCMs of the system noise are calculated via*

(48) $$P_{w_{i}w_{j}}\left(k\right)=E\left\{{\tilde{w}}_{i}^{e}\left(k|k\right){{\tilde{w}}_{j}}^{eT}\left(k|k\right)\right\}=Q_{w}\left(k\right)-\Lambda_{j}\left(k\right)S_{wv_{j}^{*}}{K_{w_{j}}}^{T}\left(k\right)+\Lambda_{i}\left(k\right)\Lambda_{j}\left(k\right)K_{w_{i}}\left(k\right){\bar{C}}_{i}^{*}P_{\varsigma_{i}\varsigma_{j}}\left(k|k-1\right){{\bar{C}}_{j}^{*}}^{T}{K_{w_{j}}}^{T}\left(k\right)\;-\Lambda_{i}\left(k\right)K_{w_{i}}\left(k\right){S_{wv_{i}^{*}}}^{T}+\Lambda_{i}\left(k\right)\Lambda_{j}\left(k\right)K_{w_{i}}\left(k\right)R_{\mathrm{ij}}{K_{w_{j}}}^{T}\left(k\right)$$

*The EECCMs between the state and the system noise are calculated via*

(49) $$P_{\varsigma_{i}w_{j}}\left(k\right)=E\left\{{\tilde{\varsigma }}_{i}^{e}\left(k|k\right){{\tilde{w}}_{j}}^{eT}\left(k|k\right)\right\}=-\left[I_{\bar{p}N}-\Lambda_{i}\left(k\right)K_{i}\left(k\right){\bar{C}}_{i}^{*}\right]P_{\varsigma_{i}\varsigma_{j}}\left(k|k-1\right){{\bar{C}}_{j}^{*}}^{T}{K_{w_{j}}}^{T}\left(k\right)\Lambda_{j}\left(k\right)\;-\Lambda_{i}\left(k\right)K_{i}\left(k\right){S_{wv_{i}^{*}}}^{T}+\Lambda_{i}\left(k\right)\Lambda_{j}\left(k\right)K_{i}\left(k\right)R_{\mathrm{ij}}{K_{w_{j}}}^{T}\left(k\right)$$

(50) $$P_{w_{i}\varsigma_{j}}\left(k\right)={P_{\varsigma_{j}w_{i}}}^{T}\left(k\right)$$

*The initial values are $P_{\varsigma_{i}\varsigma_{j}}\left(0\right)=diag\left(P_{0}1_{\left\{\theta (0)=m\right\}}\right)$ , $P_{w_{i}w_{j}}\left(0\right)=Q_{w}$ , $P_{w_{i}\varsigma_{j}}\left(0\right)=0$ , and $P_{\varsigma_{j}w_{i}}\left(0\right)=0$ .*

**Proof.**  This is similar to the derivation in [39]. Therefore, we omit it here.    □

Based on (46)–(50), the EECCMs for Systems (1) and (2) are computed via

(51) $$P_{x_{i}x_{j}}^{c}\left(k\right)=\sum_{m=1}^{N}\sum_{n=1}^{N}P_{\varsigma_{i}\varsigma_{j}}^{mn}\left(k\right)$$

(52) $$P_{w_{i}w_{j}}^{c}\left(k\right)=\sum_{m=1}^{N}\sum_{n=1}^{N}P_{w_{i}w_{j}}^{mn}\left(k\right)$$

(53) $$P_{x_{i}w_{j}}^{c}\left(k\right)=\sum_{m}^{N}\sum_{n}^{N}P_{\varsigma_{i}w_{j}}^{mn}\left(k\right)$$

(54) $$P_{w_{i}x_{j}}^{c}\left(k\right)=\sum_{m=1}^{N}\sum_{n=1}^{N}P_{w_{i}\varsigma_{j}}^{mn}\left(k\right)$$

where $P_{\varsigma_{i}\varsigma_{j}}^{mn}\left(k\right)$ is the (n,m) th sub-blocks of $P_{\varsigma_{i}\varsigma_{j}}\left(k\right)$ , $P_{w_{i}w_{j}}^{mn}\left(k\right)$ is the (n,m) th sub-blocks of $P_{w_{i}w_{j}}\left(k\right)$ , $P_{\varsigma_{i}w_{j}}^{mn}\left(k\right)$ is the (n,m) th sub-blocks of $P_{\varsigma_{i}w_{j}}\left(k\right)$ , and $P_{w_{i}\varsigma_{j}}^{mn}\left(k\right)$ is the (n,m) th sub-blocks of $P_{w_{i}\varsigma_{j}}\left(k\right)$ .

**Theorem** **4.** *Based on LFs (44) and (45), as well as the EECCMs in (51), the optimal matrix-weighted DFE can be obtained as follows [38]:*

(55) $${\hat{x}}_{0}\left(k\right)=\sum_{i=1}^{L}{\bar{A}}_{i}\left(k\right){\hat{x}}_{i}\left(k|k\right)$$

(56) $$\bar{A}\left(k\right)=\left[{\bar{A}}_{1}\left(k\right),{\bar{A}}_{2}\left(k\right),\cdots ,{\bar{A}}_{L}\left(k\right)\right]={\left(e^{T}\Sigma {\left(k\right)}^{-1}e\right)}^{-1}e^{T}\Sigma {\left(k\right)}^{-1}$$

(57) $$P_{0}\left(k\right)={\left(e^{T}\Sigma {\left(k\right)}^{-1}e\right)}^{-1}$$

*where $\bar{A}\left(k\right)$ is a matrix-weighted coefficient, ${\hat{x}}_{i}\left(k|k\right)$ is the LF of the ith sensor in the FC, $\Sigma \left(k\right)={\left(P_{x_{i}x_{j}}^{c}\left(k|k\right)\right)}_{\bar{p}L\times \bar{p}L},i,j=1,2,\cdots ,L$ , $P_{x_{i}x_{i}}^{c}\left(k|k\right)=P_{x_{i}}^{c}\left(k|k\right),i=1,2,\cdots ,L$ , and $e={\left[I_{\bar{p}},\cdots ,I_{\bar{p}}\right]}^{T}$ .*

Finally, the proposed DFE algorithm with the ET mechanism is summarized in Algorithm 1, in conjunction with the above theorems and assumptions.
**Algorithm 1** Asynchronous DFE algorithm with ET mechanism
**Initialization:**
Set the initial values x(0) , $\mu_{0}$ , $P_{0}$ , $P_{\varsigma_{i}\varsigma_{j}}\left(0\right)=diag\left(P_{0}1_{\left\{\theta (0)=m\right\}}\right)$ ,

$P_{w_{i}w_{j}}\left(0\right)=Q_{w}$ , $P_{\varsigma_{j}w_{i}}\left(0\right)=P_{w_{i}\varsigma_{j}}\left(0\right)=0$ , and *m*.
**Iterate:**
Step 1: Calculate the LF using (12), the filtering gain matrix using (15), and
the EECM using (19) in Theorem 1.
Step 2: The variance-based ET mechanism is used to determine
whether the FC receives LF data.
If $\Lambda_{i}\left(k\right)=1$ , the FC obtains LE ${\hat{\varsigma }}_{i}^{e}\left(k|k\right)$ using (34), and the EECM

$P_{\varsigma_{i}}^{e}\left(k|k\right)$ is calculated using (35), as specified in Theorem 2.
If $\Lambda_{i}\left(k\right)=0$ , ${\hat{\varsigma }}_{i}^{e}\left(k|k\right)$ and $P_{\varsigma_{i}}^{e}\left(k|k\right)$ are calculated using the one-step prediction,
which is stored in the FC; when $\Lambda_{i}\left(k-1\right)=1$ , they are computed via (36)
and (37); when $\Lambda_{i}\left(k-1\right)=0$ , they are computed via (38) and (39).
Step 3: Calculate the EECCMs $P_{\varsigma_{i}\varsigma_{j}}\left(k|k\right)$ using (47) in the FC.
Step 4: Calculate the DFE in Theorem 4 using (55)–(57).
Step 5: Set k=k+1 , and return to step 1.


## 6. Simulation Research

In this section, we use a numerical example similar to [22] to verify the proposed algorithm. While ref. [22] includes multiplicative noise in the state equation, we consider CNs and FMs in the measurement equation, an ET mechanism is introduced, and the considered system models and the model transformation methods used are different. Consequently, the proposed simulations were not directly compared with [22]. Consider the following two-dimensional, three-sensor tracking system

$$x\left(\left(k+1\right)T\right)=\Phi_{\theta (kT)}x\left(kT\right)+\Gamma_{\theta (kT)}w\left(kT\right)$$

$$y_{i}\left(n_{i}kT\right)=\beta_{i}\left(n_{i}kT\right)H_{i,\theta (n_{i}kT)}x\left(n_{i}kT\right)+G_{i,\theta (n_{i}kT)}v_{i}\left(n_{i}kT\right),i=1,2,\cdots ,L$$

where T=0.5 s , $n_{1}=1$ , $n_{2}=2$ , $n_{3}=3$ , and $x\left(k\right)={\left[\begin{array}{cc} x_{1}\left(k\right) & x_{2}\left(k\right) \end{array}\right]}^{T}$ , where $x_{1}\left(k\right)$ is the position, and $x_{2}\left(k\right)$ is the velocity. The system parameters are as follows: $\Phi_{1}=\left[\begin{array}{cc} 0.8 & 0.2T\\ 0 & 0.6 \end{array}\right]$ , $\Phi_{2}=\left[\begin{array}{cc} 1 & T\\ 0 & T \end{array}\right]$ , $\Gamma_{1}=\left[\begin{array}{c} 1.5\\ 0.6 \end{array}\right]$ , $\Gamma_{2}=\left[\begin{array}{c} 0.5T^{2}\\ T \end{array}\right]$ , $H_{1,1}=\left[\begin{array}{cc} 1 & 1 \end{array}\right]$ , $H_{2,1}=\left[\begin{array}{cc} 1 & 0.6 \end{array}\right]$ , $H_{3,1}=\left[\begin{array}{cc} 0.5 & 1 \end{array}\right]$ , $H_{1,2}=\left[\begin{array}{cc} 0.4 & 1 \end{array}\right]$ , $H_{2,2}=\left[\begin{array}{cc} 1 & 0.2 \end{array}\right]$ , $H_{3,2}=\left[\begin{array}{cc} 2 & 0.5 \end{array}\right]$ , $G_{1,1}=G_{2,1}=G_{3,1}=I_{2}$ , and $G_{1,2}=G_{2,2}=G_{3,2}=I_{2}$ .

w(k) and $v_{i}\left(n_{i}k\right)$ are simultaneously cross-related white noises, and they satisfy

$$v_{i}\left(n_{i}k\right)=\alpha_{i}w\left(n_{i}k\right)+\eta_{i}\left(n_{i}k\right),i=1,2,3$$

where $\eta_{i}\left(n_{i}k\right)\in R$ are white noises with variances $R_{\eta_{1}}=0.49$ , $R_{\eta_{2}}=0.36$ , and $R_{\eta_{1}}=0.9$ , and $\eta_{i}\left(n_{i}k\right)$ are uncorrelated with w(k) , with the variances $Q_{w}=0.64$ . We set $\alpha_{1}=0.2$ , $\alpha_{2}=0.4$ , and $\alpha_{3}=0.3$ .

The stochastic variables $\beta_{i}\left(n_{i}k\right),i=1,2,3$ represent the FMs. The probability function of $\beta_{i}\left(n_{i}k\right)$ on the interval [0,1] is $p_{t}^{i}\left(s\right)$ [13]. We set

$p_{t}^{1}\left(c\right)=\left\{\begin{array}{c} 0.2,s=0.25\\ 0.4,s=0.5\\ 0.4,s=1 \end{array}\right.$ , $p_{t}^{2}\left(c\right)=\left\{\begin{array}{c} 0.45,s=0.2\\ 0.35,s=0.4\\ 0.2,s=0.8 \end{array}\right.$ , and $p_{t}^{3}\left(c\right)=\left\{\begin{array}{c} 0.7,s=0.3\\ 0.1,s=0.6\\ 0.2,s=0.9 \end{array}\right.$

The expectations of $\beta_{i}\left(n_{i}k\right)$ are ${\bar{\beta }}_{1}=0.65$ , ${\bar{\beta }}_{21}=0.39$ , and ${\bar{\beta }}_{3}=0.45$ ; the variances are $\sigma_{1}^{2}=0.09$ , $\sigma_{2}^{2}=0.0499$ , and $\sigma_{3}^{2}=0.0585$ . $trP_{d}$ is the trace of the optimal steady-state variance with the full communication rate— $\min\left\{trP_{\varsigma_{i}}\left(k\right)-trP_{d}\right\}<m<\max\left\{trP_{\varsigma_{i}}\left(k\right)-trP_{d}\right\}$ —to highlight the simulation effect. We set m=0.9 , m=1.3 , and m=1.5 . The initial model probabilities are $\pi_{1}=0.5$ and $\pi_{2}=0.5$ , and the model transformation probability matrix $p=\left[\begin{array}{cc} 0.65 & 0.35\\ 0.35 & 0.65 \end{array}\right]$ . Moreover, we set $x\left(0\right)={\left[\begin{array}{cc} 0 & 0 \end{array}\right]}^{T}$ , $\mu_{0}={\left[\begin{array}{cc} 0 & 0 \end{array}\right]}^{T}$ , and $P_{0}=0.1I_{2}$ .

**Figure 2:**
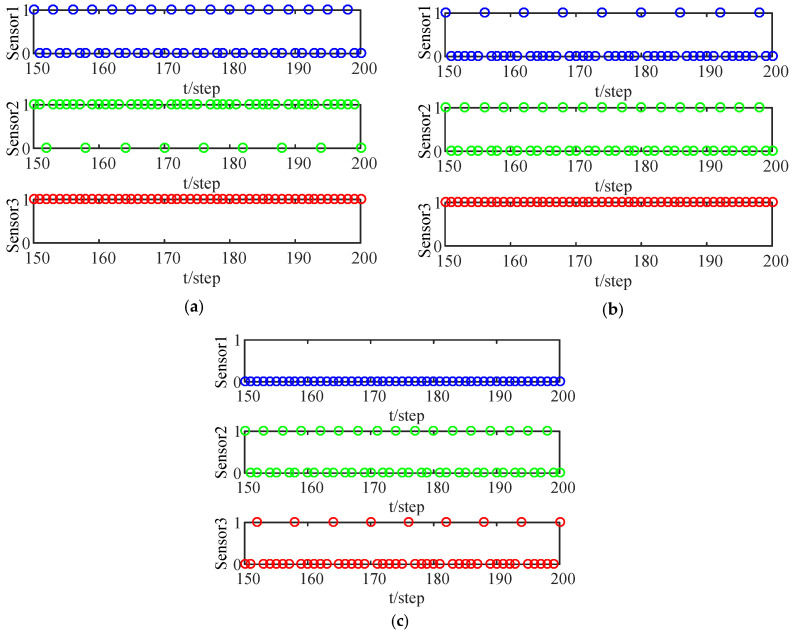
ET instants of LF with different thresholds: (**a**) ET threshold in which m=0.9 ; (**b**) ET threshold in which m=1.3 ; (**c**) ET threshold in which m=1.5 .

Figure 2 shows the transmission of the local filters from the three sensors for different thresholds $\theta_{i}$ , and parameter *m* is the main cause of the change in threshold $\theta_{i}$ . When the circle falls on 1, the FC receives the data transmitted via LF; when the circle falls on 0, the FC cannot receive the LF data. It can be seen that the larger the threshold is, the smaller the data transmission is.

**Figure 3:**
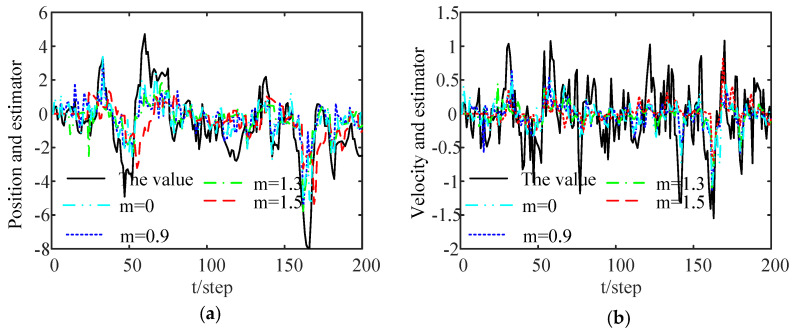
Tracking performance of the DFE based on ET mechanism: (**a**) tracking for the position; (**b**) tracking for the velocity.

Figure 3 shows a comparison of the tracking performance of the proposed DFE algorithm under different thresholds. The black solid lines denote the threshold in which m=0 . The blue lines denote the threshold in which m=0.9 . The green lines denote the threshold in which m=1.3 . The red lines denote the threshold in which m=1.5 . As shown in the figure, the algorithm can effectively track the position and velocity of the target. In addition, we also find that the tracking performance worsens with an increase in the ET thresholds.

The definition of root-mean-square error (RMSE) is given below, offering a more direct measure of algorithm effectiveness.

$$RMSE_{i,d}\left(k\right)=\sqrt{\frac{1}{M}\sum_{j=1}^{M}{\left(x_{i}^{j}\left(k\right)-{\hat{x}}_{i,d}^{j}\left(k\right)\right)}^{2}},\left(i=1,2\right)$$

where i=1,2 represents the state component, *d* represents the DFE under the ET mechanism in the FC, and *M* represents the number of Monte Carlo experiments.

**Figure 4:**
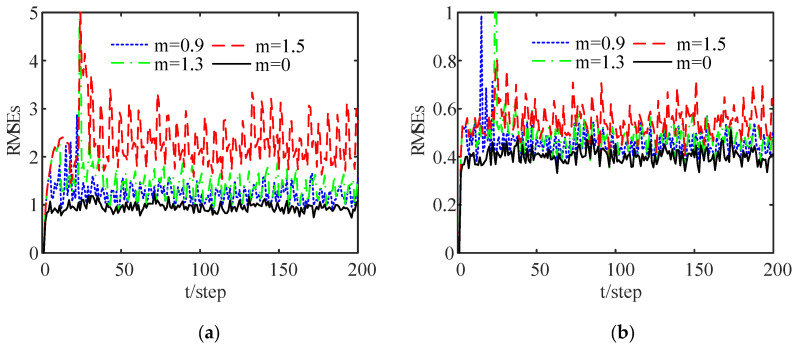
A comparison of RMSEs of the DFE with different thresholds: (**a**) RMSEs of the position; (**b**) RMSEs of the velocity.

Figure 4 shows a comparison of the RMSE curves of the DFE under different thresholds after 100 Monte Carlo runs. When m=0 , all local filters are transmitted to the fusion center, exhibiting optimal estimation accuracy. Moreover, it can be seen that the RMSE curve when m=1.5 is at the top, that is, the system performance deteriorates as the threshold increases. This indicates that the transmission of local estimates and the estimation accuracy of the proposed estimation algorithm are both reduced when the threshold is increased. As can be seen from Figure 3 and Figure 4, an appropriate threshold cannot only reduce data communication and energy consumption but also ensure the required estimation accuracy through the proposed estimation algorithm. Moreover, the FMs and Markov jump parameters can also be handled.

**Figure 5:**
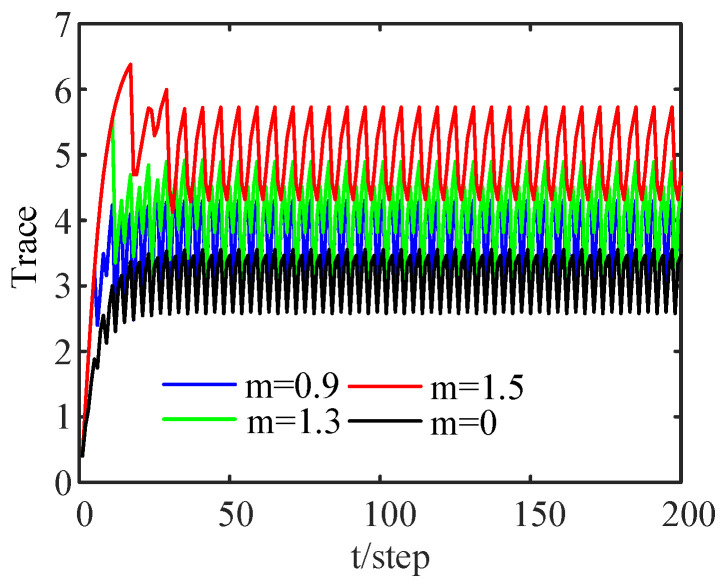
Comparison of traces of filtering error variance matrices for different thresholds.

Figure 5 shows the relationships among the traces of EECMs under different thresholds. As the threshold increases, the trace becomes larger. That is, with the increase in the threshold, the estimation accuracy is reduced. From Figure 5, it can also be seen that the fusion EECMs are periodically stable.

In summary, the proposed DFE algorithm can handle AMJSs with CNs and FMs with good estimation performance; moreover, the variance-based ET mechanism introduced between the LFs and the FC can reduce the transmission of redundant sensor data, thereby decreasing the communication bandwidth and conserving network resources.

## 7. Conclusions

In this study, a matrix-weighted DFE was studied for AMJSs with CNs and FMs based on a variance-based ET mechanism. The dummy measurement and state augmentation methods were used to establish a state–space model. The variance-based ET mechanism was introduced between the LFs and the FC to diminish redundant data transmission and reduce the communication burden. Then, a distributed fusion filter was derived based on matrix-weighted fusion criteria under the principle of linear minimum variance. A comparison of the traces of EECMs under different thresholds in numerical examples demonstrated that the proposed distributed fusion algorithm is periodically stable; however, theoretical proof remains difficult. In the future, we will analyze the stability and convergence of the proposed algorithms and generalize the results to nonlinear systems.

## Data Availability

The data presented in this study are available on request from the corresponding author. The data are not publicly available due to ownership.

## Abbreviations

The following abbreviations are used in this manuscript:

DFE	Distributed fusion estimation
FC	Fusion center
FM	Fading measurement
CN	Correlated noise
MJS	Markov jump system
AMJS	Asynchronous Markov jump system
ET	Event-triggered
LF	Local filter
EECM	Estimation error covariance matrix
EECCM	Estimation error cross-covariance matrix
RMSE	Root-mean-square error
